# Transcriptional monitoring of steady state and effects of anaerobic phases in chemostat cultures of the filamentous fungus *Trichoderma reesei*

**DOI:** 10.1186/1471-2164-7-247

**Published:** 2006-10-02

**Authors:** Jari J Rautio, Bart A Smit, Marilyn Wiebe, Merja Penttilä, Markku Saloheimo

**Affiliations:** 1 VTT Technical Research Centre of Finland, Tietotie 2, Espoo, P.O. Box 1000, 02044 VTT-Espoo, Finland; 2 Campina Innovation, Nieuwe Kanaal 7C, 6709 PA, Wageningen, The Netherlands

## Abstract

**Background:**

Chemostat cultures are commonly used in production of cellular material for systems-wide biological studies. We have used the novel TRAC (transcript analysis with aid of affinity capture) method to study expression stability of approximately 30 process relevant marker genes in chemostat cultures of the filamentous fungus *Trichoderma reesei *and its transformant expressing laccase from *Melanocarpus albomyces*. Transcriptional responses caused by transient oxygen deprivations and production of foreign protein were also studied in *T. reesei *by TRAC.

**Results:**

In cultures with good steady states, the expression of the marker genes varied less than 20% on average between sequential samples for at least 5 or 6 residence times. However, in a number of *T. reesei *cultures continuous flow did not result in a good steady state. Perturbations to the steady state were always evident at the transcriptional level, even when they were not measurable as changes in biomass or product concentrations. Both unintentional and intentional perturbations of the steady state demonstrated that a number of genes involved in growth, protein production and secretion are sensitive markers for culture disturbances. Exposure to anaerobic conditions caused strong responses at the level of gene expression, but surprisingly the cultures could regain their previous steady state quickly, even after 3 h O_2 _depletion. The main effect of producing *M. albomyces *laccase was down-regulation of the native cellulases compared with the host strain.

**Conclusion:**

This study demonstrates the usefulness of transcriptional analysis by TRAC in ensuring the quality of chemostat cultures prior to costly and laborious genome-wide analysis. In addition TRAC was shown to be an efficient tool in studying gene expression dynamics in transient conditions.

## Background

Systems-wide methods have become an important part of physiological research in industrial biotechnology, with the aim of improving industrially relevant production strains and processes, for example by identifying physiological reactions that limit metabolism or the production of proteins. Transcriptional profiling can be particularly useful, since it can reveal previously unknown, but relevant pathways [1].

Chemostat and other continuous flow cultures are the technique of choice for producing biomass for global studies, such as transcriptomic, proteomic and metabolomic profiles, since parameters such as growth rate, dissolved oxygen and nutrient concentrations can be kept constant, providing a reproducible environment and populations of cells in physiological steady state [2]. Thus reproducible physiological studies can be carried out and single parameters can be varied while others are kept constant, increasing the reliability of systems-wide datasets. Batch culture systems are also used to obtain systems-wide datasets, but because environmental conditions and growth rate are constantly changing, the interpretation of these datasets and comparison with other results is complicated [2-4].

Filamentous fungi form a notable group of cell factories that are widely exploited in the production of industrial enzymes because of their ability to produce large amounts of extracellular proteins. Production of native enzymes by the fungus *Trichoderma reesei *can exceed 100 g l^-1 ^[5]. Such levels of secretion, however, have not been reached for non-fungal recombinant proteins [6]. Use of global level 'omics' technologies, coupled with continuous chemostat cultivations, as a strategy to improve productivity is thus also emerging in the fungal research community [7,8] as the genomes of these organisms, including the one of *T. reesei *[9], become available.

Establishing chemostat cultures for filamentous fungi, however, faces particular challenges, because of their multi-nuclear, polar growth form, which introduces an inherent heterogeneity to the system. Differentiation, particularly for conidia production, may further increase culture heterogeneity for some species. In addition, fungi have a greater ability than unicellular organisms to adhere to each other, forming pellets, or to grow on solid surfaces, such as the walls of the bioreactor [10]. The filamentous growth form increases the viscosity of the culture, causing mass transfer limitations [11,12]. As with other organisms culture evolution as a result of mutations and selection occurs [13].

The quality of a chemostat steady state is generally assessed from the measurement of various process parameters such as biomass and product concentrations, CO_2 _evolution and alkali consumption rates. However, short term changes in environmental conditions, for example as a result of sample removal, and poor mixing, resulting in nutrient gradients, will have an impact on cellular physiology [14], but would not necessarily result in measurable changes in biomass related parameters. Since cells can rapidly adapt to changing conditions by transcriptional regulation [15,16], these perturbations may be affecting the transcriptome. However, the degree of stability of gene expression in continuous flow cultures has not been reported.

In these studies we have applied a novel transcriptional analysis method called TRAC (Transcript analysis with aid of affinity capture) [17] to study expression levels of a set of 30 marker genes, relative to polyA RNA content, in chemostat cultures of a *T. reesei *strain producing the laccase of *Melanocarbus albomyces *[18] and its parental strain. The TRAC method was used to monitor transcriptional steadiness and to identify disturbances in the steady state. In addition, TRAC was used to assess transcriptional responses during transient periods of oxygen deprivation and subsequent recovery and during recombinant protein production.

## Results

### Gene expression stability during steady state

The stability of several physiological parameters were monitored before and after the onset of continuous medium feeding to evaluate the steady state of chemostat cultures. Standard on-line (e.g. base consumption, dissolved O_2 _concentration and off gas concentrations for CO_2_, O_2_, N_2_) and off-line (e.g. dry weight, NH_3 _concentration, cellulase activity) measurements were used and are referred to as conventional process analyses. In addition to these conventional process analyses a novel method for rapid transcriptional profiling called TRAC [17] was used to monitor the expression of appr. 30 marker genes.

**Figure 1:**
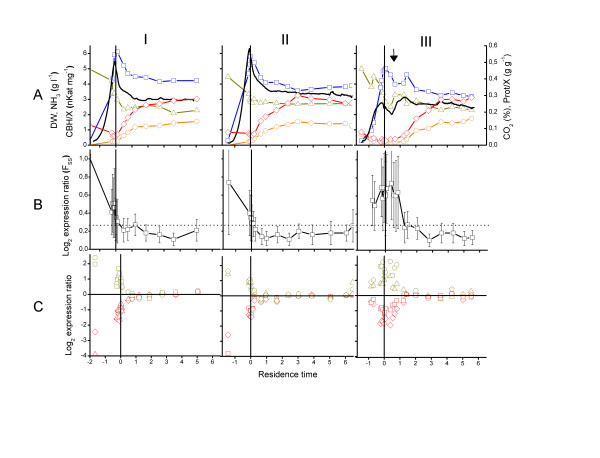
**Evaluation of marker gene expression stability during chemostat cultures with *Trihoderma reesei *RutC30**. A. Dry weight (blue squares), off gas CO_2 _concentration (black line), protein concentration (g g^-1 ^DW) (red diamonds) and specific cellobiohydrolase activity (nkat g^-1 ^DW) (orange circles) and ammonium concentration (green triangles) in chemostat cultures I – III (D = 0.03 h^-1^). In culture III arrow indicates the addition of trace mineral solution. B. Average fold-change of expression from predicted steady state of 30 marker genes (*F*_*SS*_). Error bars indicate the average deviation in *Fss *between the different marker genes. C. Expression ratios of genes showing the highest variation between batch and continuous phase (increasing expression, red symbols *cbh1*, *egl1*) and (decreasing expression, green symbols *gpd1*, *hsp70*). Residence time 0 indicates the start of the continuous growth phase. Expression levels as log_2 _expression ratios from the *F*_*ma *_predicted steady state expression level.

Stabilisation of gene expression occurred rapidly after the start of continuous medium feeding (i.e. at 0 R) in chemostats which subsequently showed good steady states (Figure 1). The genes analysed showed, on average, less than 20% variation from the most stable expression phase after approximately 0.5 R (R = 33.3 h), even though *F*_*SS *_(see Materials & Methods) showed that the average gene expression levels varied significantly between the batch and the continuous phase. In the batch phase, the genes analysed here showed on average a fold-change of 1.3 – 2.0 relative to the most stable expression phase during the chemostat culture and the average deviation in the fold change between the genes was large. The expression of secreted protein (*cbh1*, *egl1*) and folding factor genes was strongly up-regulated when the culture was in transition from batch to chemostat phase, whereas, for example, glycolysis gene *gpd1 *and heat shock protein *hsp70 *gene were strongly down-regulated (Figure 1 C).

Most chemostat cultures were lactose limited. In one batch culture, however, limitation of trace minerals (FeSO_4_, MnSO_4_, ZnSO_4_, CoCl_2_) was applied (Figure 1 III) and the transient phase between batch and chemostat growth was further disturbed by increasing the concentrations of the trace minerals to those used in other cultures, to shift the limitation from trace minerals to lactose at 0.5 R after the start of continuous medium feeding. The gene expression levels reached the same level of stability as was observed in lactose limited cultures in less than 1 R after lactose became limiting. In all cultures, the average expression levels remained constant for the next 4.5 – 5 R, with a small average deviation in F_SS _(Figure 1 I–III).

In stable chemostat cultures all measured parameters had become constant within 3 R, although different parameters required slightly different lengths of time to become constant. Stabilisation of average gene expression occurred within a similar time frame as stabilisation of CO_2 _and dry weight production, but one to two R earlier than required for secreted protein and enzyme concentrations (Figure 1).

Some marker genes showed consistent variation during the steady state phase. Expression of cellulases (*cbh1, egl1*) and folding factor (*pdi1*) was very constant in the *F*_*ma *_predicted steady state phase, with sequential measurements generally varying less than 10 % from their mean. However, variation in the expression levels for the stress related genes *gcn4*, *hsp30 *and *hsp105*, the conidiation genes *con6 *and *ccg9 *and vacuolar protease *vpa1 *was 15–20%. This level of variation was detected by TRAC with high reliability [17] and was not dependent on the fluorescence signal strength in the TRAC assay.

### Monitoring of disturbances in the steady state by TRAC

**Figure 2:**
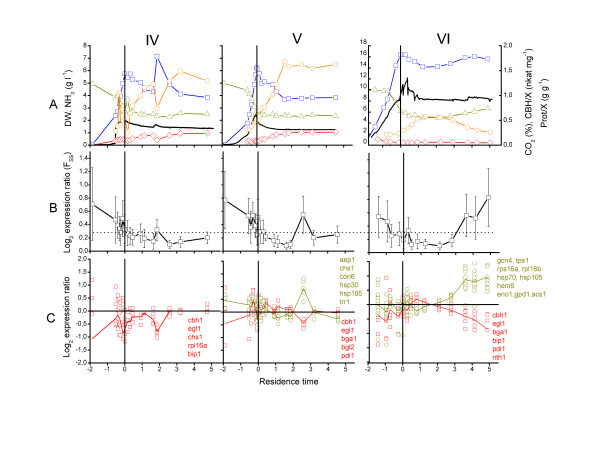
**Monitoring of disturbances in gene expression stability in chemostat cultures**. A. Dry weight (blue squares), off gas CO_2 _concentration (black line), protein concentration (g g^-1 ^DW) (red diamonds) and cellobiohydrolase activity (nkat g^-1 ^DW) (orange circles) and ammonium concentration (green triangles) in chemostat cultures IV–VI (D = 0.03 h^-1^). B. Average fold-change of expression from predicted steady state of 30 marker genes (*F*_*SS*_). Error bars indicate the average deviation in *Fss *between the different marker genes. C. Marker genes show up (green circles) or down (red squares)-regulation during the continuous phase of the cultures. Expression levels as log_2 _expression ratios from the *F*_*ma *_predicted steady state expression level

Several fungal chemostats showed some instability during the (pseudo-)steady state, in spite of the carefully controlled external parameters. Potential disturbances in the steady state and the resulting physiological instability were most sensitively detected at the level of gene expression. Figure 2 illustrates three representative chemostat cultures (IV – VI), in which instabilities occurred due to a technical disturbance (IV), an unknown effector (V) or a biological factor (VI). Instabilities were detected either as a change in gene expression and in conventionally monitored parameters (IV and VI) or as a change in gene expression only (V).

In culture IV (Figure 2) a disturbance occurred between 1.6 and 2.6 R as a result of a failure in the antifoam control. The concentration of polypropylene glycol increased 9-fold above the normal concentration causing an apparent increase in biomass concentration and a decrease in extracellular protein and enzyme concentrations. At the transcriptional level the disturbance was observed as a small increase in the average fold-change of expression of the gene set used (*F*_*SS*_), compared to the *F*_*MA *_predicted steady state. The deviation from the steady state was primarily caused by down-regulation of two main cellulases (*cbh1, egl1*), protein chaperon (*bip1*), ribosomal protein mRNA (*rpl16a*) and chitin synthase (*chs1*) (Figure 2 IV C).

In another culture (Figure 2 V), a number of genes showed a significant response to an unknown effector, while other physiological parameters were constant. Genes coding for extracellular enzymes were down-regulated together with a protein folding factor (*pdi1*), while up-regulation was observed for some stress related markers (*hsp30*, *hsp105*, *trr1*), conidation genes (*con6*, *chs1*) and alkaline extracellular protease (*aep1*).

In high density chemostat cultures, with cell dry weight 4-fold higher than in the standard culture conditions, steady states were achieved in a similar time frame to the lower biomass cultures, based on all the measured parameters. However, in cultures with high biomass a large change in expression occurred for several of the gene markers after 3 to 4 R (Figure 2 VI). The disturbance in the steady state was less evident in the conventional process measurements, although a small increase in biomass concentration and a 50% decrease in the cellobiohydrolase concentration did occur (Figure 2 VI A). The gene markers which showed down regulation after 3 R in the high biomass culture (Figure 2 VI) were the same as in other cultures which showed disturbances (Figure 2 IV, V) i.e. cellulases and folding factors. In high biomass cultures, up-regulation was consistently observed for stress related genes (*gcn4, tps1, hsp70, hsp105*), central carbohydrate metabolism genes (*gpd1, eno1, acs1*) and ribosomal protein genes.

### Monitoring transcriptional responses to anaerobic phases

We studied by marker gene expression analysis the physiological responses to fluctuation in oxygen availability and recovery of the cultures from such perturbations by exposing *T. reesei *cultures to anaerobic conditions. In several fungal chemostat cultures the steady state was disturbed after approximately 5 R by making the conditions anaerobic for 20 to 180 min. Replacing the air supply with N_2 _caused an immediate drop of dissolved and off-gas O_2 _levels to 0% (data not shown).

**Figure 3:**
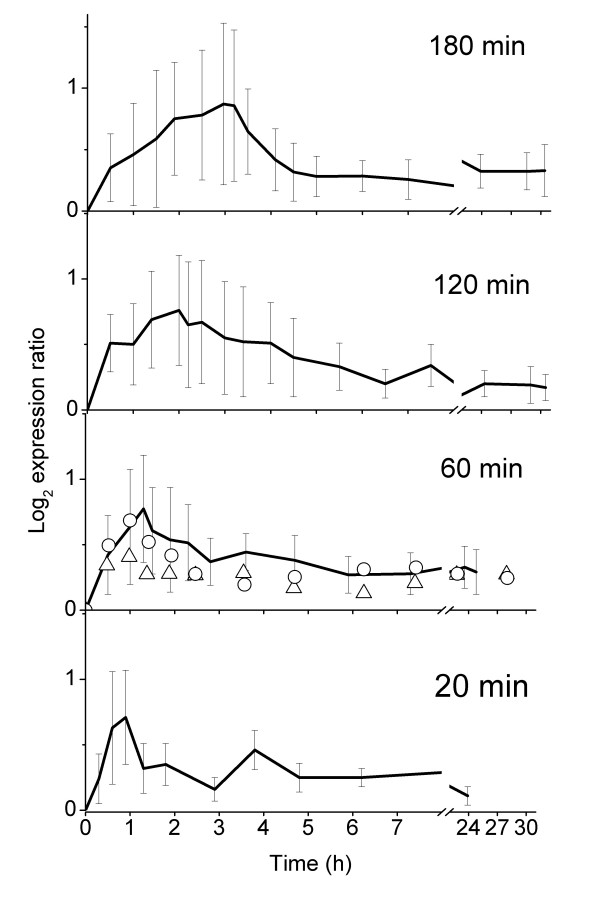
**Average responses of 30 marker genes to anaerobic phases**. In steady state phase of a chemostat culture (D = 0.03 h^-1^, pH 5) the air supply was replaced with N_2 _for 20, 60, 120 and 180 min. Curves show the average variation of the entire marker gene set (Table 1) in each sample point from the 0 h sample (initial steady state). In the figure for 60 min, circles show the corresponding average response for 60 min without either air or medium feed and triangles show the average response to 60 min without medium feed, but with air. The average variation in the responses between different marker genes is indicated by the bars.

Approximately 50 % of the marker genes showed greater than 1.5-fold change in expression level in response to anaerobic conditions. The longer the anaerobic phase, the stronger the effect for the set of genes analysed (Figure 3). However, recovery of the initial expression levels was quick after aeration of the culture was restarted. In 3 to 5 h after restoring aerobic conditions the expression of these genes showed less than 1.3-fold variation on average, from the initial steady state expression level and the expression levels remained constant for the next 20 to 30 h.

**Figure 4:**
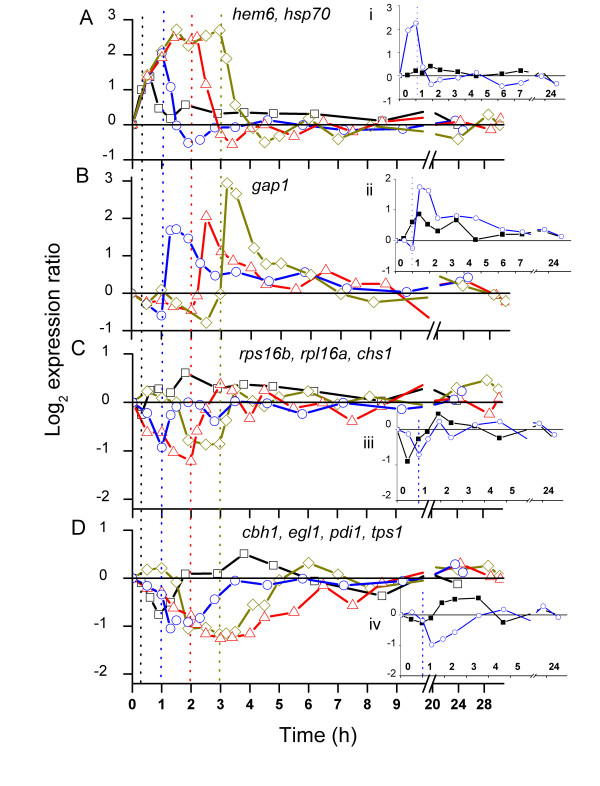
**Expression of specific marker genes in *T. reesei *during transient anaerobic phases**. Duration of anaerobic phases were 20 (black squares), 60 (blue circles), 120 (red triangles) and 180 (green diamonds) min. 0 min indicates the start of the anaerobic phase and the coloured dotted line the end of an anaerobic phase. A-D shows the average log_2 _expression ratios from the 0 min for different genes that showed similar responses during the experiment, subfigures i-iv show the average responses of the corresponding genes during 60 min without either medium or air feed (blue circles) or without medium feed (black squares).

Four different expression patterns were observed for gene markers that showed a response to sudden changes in availability of O_2 _(Figure 4 A–D). The most sensitive markers to lack of oxygen availability were *hsp70 *and *hem6 *(Figure 4 A). Both of these genes were strongly induced during anaerobiosis, and immediately repressed after aerobic conditions were restored, returning the previous, aerobic expression levels within less than 1 h.

The amino acid permease gene *gap1 *showed little or no response to the anaerobic conditions, but showed 5–8-fold induction 15 min after restoring aeration (Figure 4 B). The genes *gpd1 *and *acs1*, involved in central carbohydrate metabolism, also showed induced expression during the transition from anaerobic to aerobic conditions (data not shown). These genes only returned to the previous steady state after 5–6 h. When cultures were starved of carbon for 60 min, *gap1 *was induced. It was then down-regulated after medium feed was restarted (Figure 4 B ii).

Ribosomal protein and chitin synthase gene (*chs1*) expression levels decreased during anaerobiosis and also during lactose starvation. The average mRNA half life (T_1/2_) for these genes was shorter during lactose starvation (33 min) than anaerobic conditions (41 min) with ribosomal protein mRNAs having 15–40% shorter T_1/2 _than *chs1*. Up-regulation to previous steady state expression level occurred after the restoration of aeration (Figure 4 C).

The genes for secreted cellulases, *cbh1 *and *egl1*, and the protein folding factor gene, *pdi1*, which had been observed to be down-regulated in various process disturbances (Figure 2), were also down-regulated (by appr. 2-fold) by anaerobiosis, along with the trehalose-6-phosphate synthase (*tps1*) gene (Figure 4 D). These genes slowly recovered their previous expression levels after restoration of aerobic conditions. The specific activity of CBH and EG (nkat g^-1 ^dry weight) decreased 15 to 30% in anaerobic conditions, but increased to the previous activities once aerobic conditions were restored (data not shown). In contrast, during 60 min lactose starvation expression of these genes did not change and they were up-regulated after medium feed was restarted. (Figure 4 D iv).

To determine whether the responses described above were specific for anaerobiosis and subsequent restoration of aeration or due to the accumulation of substrate or other metabolites during the anaerobiosis, chemostat cultures were also carried out in which the medium feed was stopped with or without simultaneous termination of air feed (see Materials & Methods). The effect of anaerobic conditions was the same, regardless of whether the culture was starved of lactose or had excess of lactose (Figure 4 A i – D iv). The transcriptional response to oxygen starvation was different than the response to lactose starvation for all genes except the ribosomal protein and chitin synthase genes (Figure 4 C iii), and thus for the other genes observed response was specific for lack of oxygen.

### Comparison of marker gene expression between a recombinant and the parental strain

**Figure 5:**
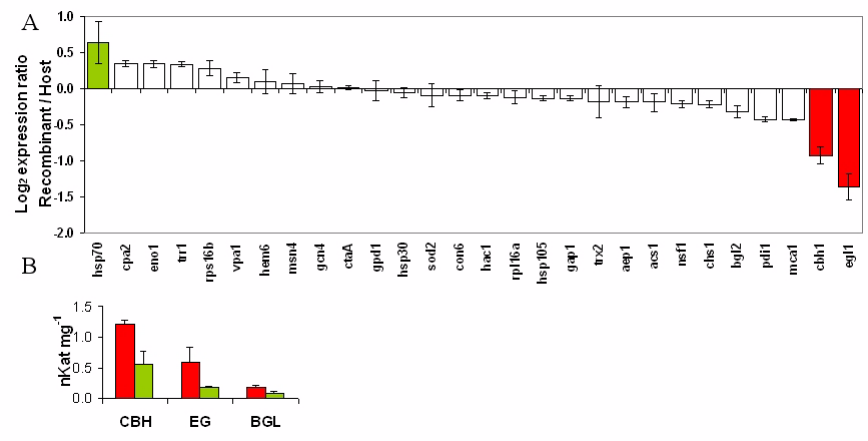
**Comparison of marker gene expression and cellulose production between recombinant laccase producing and parental strains**. A. Log_2 _ratios of gene expression between recombinant and parental strains in chemostat cultures (D = 0.03 h^-1^). Error bars indicate the standard deviation between triplicate cultures. Genes showing higher expression ratios than 1.5-fold are marked as green (higher in recombinant strain) or red (higher in parental strain) B. Comparison of specific production levels (nKat mg^-1 ^DW) of three secreted hydrolases (CBH, EG and BGL) between parental (red) and recombinant (green) strains.

One of the aims in our studies was to address the physiological effects of foreign protein production to the cell. For focused and genome-wide expression comparison, using transcriptional stability (<20% variation from *F*_*MA *_predicted steady state) as a decisive criterion, the most stable cultures among those cultures assessed as being in steady state based on conventional process analyses were selected. Figure 5 gives a comparison of marker gene expression between *T. reesei *RutC-30 and the *M. albomyces *laccase producing strain during steady state in lactose limited chemostat cultures at D = 0.03 h^-1^. Results of the genome-wide analysis will be published elsewhere.

Expression of each marker gene was averaged over 5 samples during the *F*_*MA*_-predicted steady state from triplicate cultures. Out of approximately 30 marker genes, only 3 showed significant difference in expression (>1.5-fold difference) between the two strains (Figure 5 A). Gene expression of native secreted cellulases *cbh1 *and *egl1 *were down-regulated in the recombinant strain by 1.9 and 2.6-fold respectively. Another cellulase, intracellular β-glucosidase 2 (*bgl2*) showed a smaller 1.3-fold down regulation in the recombinant laccase producing strain, compared to the host strain. The reduced expression levels corresponded with the observed 2.1- and 3.3-fold lower specific extracellular activities for CBH and EG, respectively (Figure 5 B). The unfolded protein response (UPR) (*pdi1*, *hac1*) and trafficking related (*nsf1*) genes were moderately down-regulated in the recombinant strain.

Only one gene in the gene set analysed, coding for protein chaperon *hsp70*, was up-regulated in the recombinant strain. The expression level of this gene showed considerable variation between the triplicate cultures of the same strain, and the observed variation may therefore be partly culture dependent.

## Discussion

In this study we addressed the quality of a large number of chemostat cultures performed with *Trichoderma reesei*. This was done by conventional process analysis and by a novel transcriptional analysis method TRAC that allows measurement of multiple mRNA levels in a large number of samples.

To achieve a physiological steady state in chemostat cultures with filamentous fungi can be considered as challenging due to various factors causing inhomogeneity to the culture (wall growth, viscosity, presence of metabolically active and inactive biomass *etc*.). Consistent with this, only in some of the *T. reesei *chemostat cultures performed in this work good physiological steady states were observed, based on the stability of all measured parameters (conventional and mRNA). In these cultures, monitoring of marker genes showed that most transcripts had reached their steady state expression level (<20% deviation from the predicted steady state) within less than 1 generation of the onset of continuous medium flow and that expression levels remained constant for the following 5 to 6 generations or until the culture was terminated or deliberately perturbed. Cultures, chosen by TRAC for comparison of the two strains, showed a high degree of reproducibility during the steady state (Figure 5). The average variation between parallel TRAC measurements has been show to be approximately 9% [17].

Steady states were not obtained in all cultures, nor were the reasons for this always apparent. Instabilities were always evident at the level of transcription, but were not always detectable in the conventional process analyses. The changes observed at the transcriptional level indicated that some undetected perturbation in the environmental conditions had occurred. Microorganisms are able to rapidly adjust their internal physiology to variable conditions [15], and adjustment of genetic expression is expected to be among the earliest detected changes in cellular physiology in response to environmental perturbations. Monitoring of relevant marker genes will also give an indication as to the source of perturbation.

In chemostat cultures with *T. reesei*, the most sensitive markers for process disturbances were the genes coding for hydrolytic enzymes (*cbh1, egl1, bgl2, bga1*), which were consistently down-regulated in response to various factors like high concentrations of polypropylene glycol, lack of aeration or high cell density stress. This observation has special importance, since these enzymes are the major products of *T. reesei *in industrial production, and the *cbh1 *promoter is widely used to drive expression of foreign proteins. The down-regulation of secreted enzyme genes was accompanied by down-regulation of UPR-related folding factors (*pdi1, bip1*). The co-regulation of these two gene groups has been observed earlier [19]. Down-regulation of *cbh1 *and *egl1 *was also reflected in subsequent reduction in the corresponding enzyme activity (Figures 2 and 5). The *T. reesei *cultures clearly respond to several stress conditions at the expense of protein production and secretion.

Expression of genes coding for conidiation related (*con6, chs1*) and ribosomal proteins (*rps16a*, *rpl16b*) was also sensitive to process disturbances. Reduced transcription of ribosomal protein mRNAs has also been observed in yeast under various stress conditions, such as nutrient starvation, protein secretion stress and osmotic stress [16]. Since ribosomal RNA genes make up a considerable fraction of the entire genome, the reduced synthesis of rRNA and ribosomal protein transcripts and of the ribosomes may help to save energy while the cells adapt to changing conditions [20]. In rapidly growing cells the expression of ribosomal protein mRNA is, by contrast, strongly induced [21].

*Con6 *encodes a short protein that is homologous to *Neurospora crassa *conidiation specific gene 6. *N. crassa con6 *is expressed during the formation of asexual conidia (spores), but is not expressed in mycelium [22]. Chitin synthase (*chs1*) has a role in cell wall biogenesis and is required for formation of conidia in *N. crassa *[23]. These conidiation related genes showed relatively high instability during non-disturbed as well as disturbed chemostat cultures. High levels of conidiation were observed in several of the cultures that did not attain steady state (data not shown) and variation in the expression of these genes during steady states could reflect a need to continually adjust the balance between filamentous growth and conidium formation, in order to maintain constant biomass in the chemostat.

In high cell density *T. reesei *chemostat cultures after 3 to 4 R several of the marker genes showed a significant fold-change, relative to their expression levels during the most stable expression phase (Figure 2 VI). The up-regulation of the trehalose-6-phosphate synthase gene *tps1 *and repression of the trehalase gene *nth1 *suggested that trehalose synthesis occurred in the high biomass *T. reesei *cultures, as has been observed in *S. cerevisiae *when grown in high substrate concentrations [24]. Up-regulation of the carhohydrate metabolism genes (*acs1*, *gpd1*, *eno1*) in *T. reesei *indicated elevated glycolytic flux and higher energy demand [24,25]. *Hem6 *and/or *hsp70 *genes were induced after 3 R in the high cell density cultures (Figure 2 VI), possibly indicating limitation in oxygen availability, since regulation of these genes was also observed to be oxygen dependent (Figure 5 A). The increase in transcription factor *gcn4 *mRNA could suggest an amino acid starvation response [26]. Unexpected increase in biomass concentration was observed, which could be connected to up-regulation of ribosomal protein mRNAs (Figure 2 VI).

When the steady states of the fungal cultures were deliberately disturbed by exposing them to anaerobic conditions for 0.3 – 3 h, the return of transcript levels of affected genes to their previous level was surprisingly rapid once aeration of the culture was restarted. Within 5 h of restarting aeration the expression of the genes were at the previous steady state levels. Although *T. reesei *is not able to grow in anaerobic conditions, it sustained metabolic activity surprisingly well without oxygen. Short term deprivation of oxygen level to the culture did trigger physiological responses, but *T. reesei *recovered in relatively short time from even 3 h of total anaerobiosis (Figure 4) and no signs of cell lysis were observed (data not shown). Bonaccorsi *et al*. [27] observed a similar restoration of gene expression in *T. reesei *QM9414 within 2 h following a 2 h period in anaerobic conditions at D = 0.01 h^-1 ^with glucose as the carbon source. Bonaccorsi *et al*. [27] monitored 2000 genes, 19.6 % of which were affected by oxygen concentration, but did not confirm steady state expression levels, as was possible here using TRAC with a smaller number of genes. Along with medium composition, the exchange of gasses is a critical parameter in industrial fermentation processes [11,28]. In large scale fermentations with a thick filamentous slurry, inhomogeneity of the system leads to local limitations in oxygen supply [12]. As with other process disturbances, the cellulase genes were repressed during oxygen deficiency (Figure 4 D), as has previously been observed for *cbh1 *and *egl1 *[29]. This is a crucial factor considering the productivity of the *T. reesei *production process.

Genes coding for coproporphyrinogen III oxidase (*hem6*) and heat shock protein 70 (*hsp70*) were the most sensitive markers among the genes assessed here for availability of O_2_. Coproporphyrinogen III oxidase is involved in heme biosynthesis. Heme functions in yeast as an oxygen sensor by regulating the activity of transcription factor Hap1. Heme permits Hap1 to be released from a high-molecular-weight complex (HMC) in the presence of oxygen, and Hap1 subsequently activates transcription of aerobic genes and represses expression of anaerobic genes by repressing Rox1 activity [30-32]. The absence of oxygen induces the expression of half of the heme biosynthesis genes in *S. cerevisiae*, with the *hem6 *homologue HEM13 showing the strongest up-regulation, even though heme cannot be synthesised in anaerobic conditions [33]. In *S. cerevisiae *Ssa type Hsp70 proteins have been shown to be a part of the HMC [34] and to be involved in activation of anaerobic genes via the binding of Hap1p in the absence of heme. The O_2 _dependent regulation of the *T. reesei *Ssa homologue *hsp70 *shown here, may thus reflect its role in the regulation of HAP1 activity.

Inhibition of growth by either lack of lactose or of oxygen resulted in down-regulation of ribosomal protein and chitin synthase genes as expected. Rapid up-regulation to previous expression level was observed after restoring previous steady state conditions. Yeast ribosomal protein mRNAs decline in mild heat shock with T_1/2 _of 5–7 min [35], which was much faster than the turn-over times observed in this work for two *T. reesei *ribosomal protein mRNAs under lactose starvation (T_1/2 _= 28 min) or anaerobiosis (T_1/2 _= 37 min). The rapid increase (doubling in 60 min) in *chs1 *and ribosomal protein mRNA levels when culture again became aerobic was consistent with the observation that cell density had not decreased during the short term anaerobiosis. The rapid and strong up-regulation of aminoacid permease may also be part of the quick adaptation of the culture to growth conditions (Figure 4 B).

TRAC analysis demonstrated that the expression levels of the marker genes were very similar for RutC-30 and the strain producing recombinant laccase, with only few exceptions (Figure 5). Down-regulation of native endo- and exoglucanases (*cbh1, egl1*) was observed in the recombinant strain, corresponding to the lower specific production of these enzymes (Figure 5 B). This could be due to repression of the genes by the RESS mechanism (repression under secretion stress, [8]), even though no unfolded protein response (UPR, [36]) was observed. Another possible explanation could be that the cellulase genes were down-regulated due to titration of their activating transcription factors [37]. This idea, however, is questioned by the fact that the laccase expression construct is present in the analysed strain as a single copy (data not shown).

Transcriptional monitoring of chemostat cultures is beneficial prior to costly and laborious systems-wide analyses in order to increase their reliability by ensuring that samples from only reproducible cultures are analysed. In addition to offering a high degree of reproducibility, TRAC also has the advantage of allowing simultaneous analysis of a large number of samples, in a cost effective, time efficient manner. Therefore, in addition to monitoring of transcriptional stability, TRAC is well suited for analysis of gene expression dynamics in experiments involving transients.

## Conclusion

We have analysed the expression stability of approximately 30 marker genes in a large number of chemostat cultures of the filamentous fungus *Trichoderma reesei*, using the novel transcriptional analysis method TRAC. We have demonstrated that gene expression levels were stable in those chemostat cultures showing good physiological steady states. Instabilities in the chemostat cultures were always detected at the level of transcription, but were not always detectable by conventional process analyses. Various disturbances, as well as expression of a recombinant laccase, were particularly manifest by the down-regulation of genes coding for the hydrolytic enzymes, which are the major industrial products of *T. reesei*. Deliberate exposure of the *T. reesei *chemostat cultures to transient anaerobic phases, revealed that the fungus was able regain previous steady state expression levels rapidly once aeration was restarted. This study demonstrates the usefulness of TRAC in transcriptional analysis of transient conditions, as well as ensuring the quality of chemostat cultures prior to systems-wide analysis.

## Methods

### Strain and medium

*Trichoderma reesei *Rut-C30 [38] and its transformant pLLK13/295 producing *Melanocarpus albomyces *laccase [18] were used in these studies.

The medium contained: KH_2_PO_4 _15 g l^-1^, (NH_4_)_2_SO_4 _5 g l^-1^, CaCl_2_·2H_2_O 0.6 g l^-1^, MgSO_4_·6H_2_O 0.6 g l^-1^, CuSO_4_·5H_2_O 30 mg l^-1^, FeSO_4_·5H_2_O 5 mg l^-1^, MnSO_4_·H_2_O 1.6 mg l^-1^, ZnSO_4_·7H_2_O 1.4 mg l^-1^, CoCl_2_·6H_2_O 3.7 mg l^-1 ^and lactose 10, 20 or 40 g L^-1^. Salt concentrations in cultures containing 40 g lactose l^-1 ^were increased to: KH_2_PO_4 _15 g l^-1^, (NH_4_)_2_SO_4 _12.5 g l^-1^, CaCl_2_·2H_2_O 1.5 g l^-1^, MgSO_4_·6H_2_O 1.5 g l^-1^, CuSO_4_·5H_2_O 30 mg l^-1^, FeSO_4_·5H_2_O 12.5 mg l^-1^, MnSO_4_·H_2_O 4.0 mg l^-1^, ZnSO_4_·7H_2_O 5.6 mg l^-1^, CoCl_2_·6H_2_O 14.8 mg l^-1^

### Cultivation conditions

Inoculum was prepared by transferring 1 × 10^8 ^spores into a 1.5 l conical flask containing 500 ml growth medium (lactose 20 g l^-1^). The culture was grown for approximately 72 h at 28°C with shaking at 200 rpm and then 200 or 500 ml were transferred to a bioreactor (Braun Biostat CT2-DCU3 or CT5-2, B. Braun Biotech International GmbH, Meisungen, Germany) along with sufficient medium to give total final volumes of 2 or 5 l respectively. Cultivations in the bioreactors were carried out at 28°C, with aeration of 0.5 vvm (volumes of air per volume of liquid per minute) and stirring at 800 rpm. The pH was maintained at 4.8 ± 0.1 using 1 M NaOH. Approximately 0.2% v/v (final concentration) polypropylene glycol (mixed molecular weights; [39]) was used as an antifoam agent. Nitrogen, oxygen and carbon dioxide were monitored online by measuring peak areas at *M/z *= 24, 32 and 44 with an OmniStar mass specrometer (Pfeiffer Vacuum, Germany).

For lactose limited chemostat culture, the feed was started immediately after CO_2 _production stopped at the end of the batch phase, approximately 50 h after inoculation. The feed was supplied at constant rate to maintain a dilution rate of 0.03 h^-1 ^for 5 to 6 residence times (R).

To study the effect of anaerobic conditions, air supply to the fermentor was replaced by N_2 _for 20, 60 (in duplicate), 120 or 180 min in chemostat cultures after 5 R when the cultures were in steady state. It was assumed that during anaerobic conditions the fungus would not be able to metabolise all the fed lactose and thus substrate would accumulate. To study which of the transcriptional responses observed were specific for the transition from anaerobic to aerobic conditions and which resulted from the increased concentration of substrate or other metabolites, the supply of growth medium was stopped for a 60 min period in one culture and in another culture medium supply was stopped simultaneously with replacing the air flow by N_2_, also for a 60 min period.

Samples (20 – 50 ml) were withdrawn from the fermentor via a sterilisable port. Two times five mL aliquots were immediately filtered using glass-fibre filter disks (Whatman GF/B 47 mm ∅, Kent, UK). The biomass was immediately washed with 20 ml sterile, demineralised water, after which the biomass was removed to liquid nitrogen and stored at -80°C for further analysis. The filtrate was collected for protein, sugar and enzyme analyses. These samples were also frozen in liquid nitrogen and stored at -80°C until analysed. Samples were collected most frequently some hours before and after the transient from batch to continuous phase and 2–3 times per day during the continuous phase. Samples for transcriptional analysis during and after the anaerobic conditions were collected every 15 to 60 min for the first 8 h and every 2 to 6 h until 30 h.

### Analyses

#### Dry weight

For determination of dry weight, two sample aliquots were weighed and collected on pre-weighed filter disks (Whatman GF/B 25 mm ∅). The biomass was washed with approximately 50 ml demineralised water and dried overnight in an oven at 110°C, before the disks were re-weighed.

#### Enzyme activities

The activities of the cellulolytic enzymes were determined using a Cobas-mira (Roche, Switzerland) autoanalyser. For measurement of total cellulolytic (i.e. cellobiohydrolase (CBH), endoglucanase (EG) and β-glucosidase (BGL)) activity 10 μl sample was added to 100 μl reagent containing 100 mM sodiumcitrate buffer pH = 5.0 and 6.0 mM p-Nitrophenyl-β-D-lactopyranoside (Sigma, St. Louis, MO). After 16 min incubation at 37°C, 50 μl 1.5 M sodium carbonate was added to raise the pH above 10 and the absorbance was read at 405 nm. Activity was determined from the amount of p-Nitrophenol released, as determinded from a calibration curve made under the same reaction conditions, and is reported as nkat mg^-1 ^biomass. Individual activities of CBH, EG and BGL were estimated by adding 10 mM cellobiose (inhibits mainly CBH) or 100 mM glucose (inhibits BGL) to the assay.

#### Protein and ammonium

Protein was assayed by mixing 10 μl sample with 200 μl reaction mixture (Biorad, Hercules, CA) in a microtiter plate. After 15 minutes incubation at 20°C, absorbance was measured at 595 nm. Samples were measured in duplicate and protein concentration was determined from a Bovine Serum Albumin (Biorad) calibration curve.

The ammonium concentration was determined using the Roche ammoniak test kit (Basel, Switzerland), according to the instructions of the manufacturer, adapted for automated analysis with the Cobas-mira (Roche).

### Transcriptional analysis by TRAC

Transcriptional analysis was performed with the TRAC assay as described in Rautio et al. [17]. 0.3 – 1 mg (wet weight) of lysed mycelium, containing 50 – 200 ng of polyA mRNA, was added to each hybridisation reaction with 4 pmol biotinylated oligo(dT) capture probe and 1 pmol of each 6-FAM-labeled detection probe. The hybridizations were carried out in 96-well PCR plates (ABgene, Epsom, UK) at 60°C for 30–40 min with shaking at 600 rpm (Thermomixer Comfort, Eppendorf, Hamburg, Germany) in a total volume of 100 μl, containing 5 × SSC, 0.2% SDS, 1 × Denhardt solution (0.02% (w/v) Ficoll, 0.02% (w/v) polyvinyl pyrrolidone, 0.02% (w/v) BSA) and 3% (w/v) dextran sulfate.

The steps following hybridization, including affinity capture, washing and elution, were automated with a magnetic bead particle processor KingFisher 96 (Thermo Electron, Vantaa, Finland) in 96-well plates, as follows: 1) affinity capture of hybridized RNA targets to 50 μg of streptavidin-coated MyOne DynaBeads (Dynal, Oslo, Norway) at room temperature for 30 min, 2) washing of the beads two times for 1.5 min in 150 μl of 1 × SSC, 0.1% (w/v) SDS at room temperature, 3) washing twice for 1.5 min in 150 μl of 0.5 × SSC, 0.1% (w/v) SDS at RT, 4) washing once for 1.5 min in 150 μl of 0.1 × SSC, 0.1% (w/v) SDS at RT and 5) elution of probes to 10 μl deionised formamide (Sigma) for 20 min at 37°C.

The eluents were analyzed by capillary electrophoresis with an ABI PRISM 310 Genetic Analyzer (Applied Biosystems, Foster City, CA). In order to compare individual samples and to calibrate the separation of the detection probes by size, GeneScan-120LIZ size standard (Applied Biosystems) was added to each sample. The identity of the probes was determined by the migration and the quantity by the peak area.

Total polyA RNA quantification from prepared lysates was performed with the above TRAC protocol without addition of detection probes. The final elution of polyA mRNA was performed in 50 μl DMPC treated water. RNA concentration in the eluent was quantified with a RiboGreen RNA quantitation kit (Molecular Probes, Leiden, the Netherlands).

### Probe pools

The detection probe oligonucleotides, labelled at the 3' and 5' ends with 6-carboxy fluorescein (6-FAM), were synthesized by Thermo Electron (Ulm, Germany). The biotinylated Oligo(dT) capture probe was from Promega. The HPLC-purified oligonucleotide detection probes were organised into three pools (Table 1). Oligonucleotide probes were designed using mathematical algorithms presented in Kivioja *et al*. [40]. Criteria used in probe selection were the following: melting temperature, T_m_, limits 60 – 70°C, GC% limits 38 – 62, maximum repeat size 15 nt, maximum similarity e = 0.8, maximum free energy change in hybridisation [41] > -15 kcal/mol and minimum target energy change, A_c_, [41] < -10 kcal/mol. Tms were calculated with the nearest neighbouring method according to le Novére [42] using 10 nM nucleic acid and 750 mM salt concentrations.

The marker genes used are involved in various cellular functions, including carbohydrate metabolism, protein production and degradation, transport, growth, conidiation and stress protection against various factors such as unfolded proteins and reactive oxygen species (Table 1).

### Calculation of gene expression stability in chemostat cultures

The fluorescence signal intensity measured for gene specific probes in capillary electrophoresis is normalised using the total polyA mRNA amount in each hybridisation reaction. To estimate the stability of the expression of the set of gene markers in the different phases of the chemostat cultures we have calculated the moving average of fold change (*F*_*ma*_)(equation 1). *F*_*ma *_predicts the time interval in which the culture was in steady state based on the average deviation in the expression of the marker genes from their mean expression level between 5 sequential measurements being at its lowest. *F*_*ma *_is calculated over all groups of 5 sequential measurements throughout the experiment.

Fma=15N∑k=1N∑i=04|log⁡2(5xk(t+i)∑j=04xk(t+j))|     1)
 MathType@MTEF@5@5@+=feaafiart1ev1aaatCvAUfKttLearuWrP9MDH5MBPbIqV92AaeXatLxBI9gBaebbnrfifHhDYfgasaacH8akY=wiFfYdH8Gipec8Eeeu0xXdbba9frFj0=OqFfea0dXdd9vqai=hGuQ8kuc9pgc9s8qqaq=dirpe0xb9q8qiLsFr0=vr0=vr0dc8meaabaqaciaacaGaaeqabaqabeGadaaakeaacqWGgbGrdaWgaaWcbaGaemyBa0Maemyyaegabeaakiabg2da9maalaaabaGaeGymaedabaGaeGynauJaemOta4eaamaaqahabaWaaabCaeaadaabdiqaaiGbcYgaSjabc+gaVjabcEgaNnaaBaaaleaacqaIYaGmaeqaaOWaaeWaceaadaWcaaqaaiabiwda1iabdIha4naaBaaaleaacqWGRbWAaeqaaOWaaeWaceaacqWG0baDcqGHRaWkcqWGPbqAaiaawIcacaGLPaaaaeaadaaeWbqaaiabdIha4naaBaaaleaacqWGRbWAaeqaaOWaaeWaceaacqWG0baDcqGHRaWkcqWGQbGAaiaawIcacaGLPaaaaSqaaiabdQgaQjabg2da9iabicdaWaqaaiabisda0aqdcqGHris5aaaaaOGaayjkaiaawMcaaaGaay5bSlaawIa7aaWcbaGaemyAaKMaeyypa0JaeGimaadabaGaeGinaqdaniabggHiLdaaleaacqWGRbWAcqGH9aqpcqaIXaqmaeaacqWGobGta0GaeyyeIuoakiaaxMaacaWLjaGaeGymaeJaeiykaKcaaa@6743@

where,

*x*_*k*_*(t) *= Expression value of gene k (k = 1, ...., N) at time point t

*t *= starting time point of 5 sequential measurements

Average relative deviation of the marker gene set from the *F*_*ma *_predicted steady state at any given time point is given by *F*_*SS *_(Average fold change of expression from steady state) (equation 2).

Fss=1N∑k=1N|log⁡2(5xk(t)∑j=04xk(ts+j))|     2)
 MathType@MTEF@5@5@+=feaafiart1ev1aaatCvAUfKttLearuWrP9MDH5MBPbIqV92AaeXatLxBI9gBaebbnrfifHhDYfgasaacH8akY=wiFfYdH8Gipec8Eeeu0xXdbba9frFj0=OqFfea0dXdd9vqai=hGuQ8kuc9pgc9s8qqaq=dirpe0xb9q8qiLsFr0=vr0=vr0dc8meaabaqaciaacaGaaeqabaqabeGadaaakeaacqWGgbGrdaWgaaWcbaGaem4CamNaem4Camhabeaakiabg2da9maalaaabaGaeGymaedabaGaemOta4eaamaaqahabaWaaqWaceaacyGGSbaBcqGGVbWBcqGGNbWzdaWgaaWcbaGaeGOmaidabeaakmaabmGabaWaaSaaaeaacqaI1aqncqWG4baEdaWgaaWcbaGaem4AaSgabeaakiabcIcaOiabdsha0jabcMcaPaqaamaaqahabaGaemiEaG3aaSbaaSqaaiabdUgaRbqabaGcdaqadiqaaiabdsha0naaBaaaleaacqWGZbWCaeqaaOGaey4kaSIaemOAaOgacaGLOaGaayzkaaaaleaacqWGQbGAcqGH9aqpcqaIWaamaeaacqaI0aana0GaeyyeIuoaaaaakiaawIcacaGLPaaaaiaawEa7caGLiWoaaSqaaiabdUgaRjabg2da9iabigdaXaqaaiabd6eaobqdcqGHris5aOGaaCzcaiaaxMaacqaIYaGmcqGGPaqkaaa@5F85@

where,

*t*_*s *_= starting time point of *F*_*ma *_predicted steady state

## Authors' contributions

JJR carried out the TRAC analysis, all data analysis and drafted the manuscript. BAS designed and carried out all bioreactor cultivations and growth and protein production analyses and helped to draft the manuscript. MW participated in the design of the study and helped to draft the manuscript. MP participated in the conception, design and coordination of the study. MS conceived of the study, and participated in its design and coordination and helped to draft the manuscript. All authors read and approved the final manuscript.

## Figures and Tables

**Table 1:** Marker genes used in the TRAC analysis.

**Gene name**	**Length nt**	**ORF no**.	**Function**	**Sequence**
**Pool1**				
*cbh1*	25	22421	cellobiohydrolyase	CATTCTGGACATAGTATCGGTTGAT
*egl1*	27	42363	endoglucanase	CGGACTTTGTACACTTGTAGGTTGTCA
*sod2*	29	42675	Superoxide dismutase	TTGATGACGTCCCAGATGGCGCTGAAGTA
*pdi1*	33	45146	protein disulfide isomerase	GGTCAAAGGGGAACTTGAGGTTCTTCTCAATGT
*gap1*	33	43090	general amino acid transporter	TGATACTTCCAGGCATTGCGGAATCGGATGTGG
*axp1*	33	21826	acidic extracellular protease	AAGTTGAAGGTGGCATCCTTGATGTTTGCTTTG
*trx2*	39	9865	thioredoxin protein	CAAACTTGACAAAGTGGACCTTGTCCTTGAACTCTGCGT
*ccg9*	39	43571	Clock controlled gene, cell wall biogenensis	AAACTTTGACTTCGAACCCTTCATACGTCGACAGTTGAA
*bgl2*	41	42449	β-glucosidase	CGTTATAGTACTTGACCCTGAAGTCATCTTCGAGAATCTTC
*bga1*	43	45191	β-galactosidase	TTTGGAATGCCAATGATCTCGAGCGGCGTTGTGACGTTGAAAT
**Pool2**				
*mca1*	25	20144	metacaspase, cysteine protease, apoptosis	AATACCCTGCGTGGAGTAGATGTAC
*bip1*	27	42955	protein chaperon	AGGGGGTTGACGTCCATGAGAACAATG
*aep1*	25	46483	alkaline extracellular protease	CATGGAGGTGCCGCTAATCGTGTTT
*vpa1*	27	44744	vacuolar protease A	GTGATGTCGGGGAGGGAATCACGCTTG
*hsp30*	31	29985	heat shock protein	GTACTTTGCGTTGTCGGTAGGCTTGTTGCTG
*con6*	31	35051	ligth induced conidiation gene	TGCTTAGCGTTTTCCTTTGCTTCCTCCGACA
*trr1*	31	45052	thioredoxin reductase	AATGACGAAGAGGGGCTTGTTGCGGAAGATG
*gcn4*	37	37844	transcription factor	TGAAGAAGACGATCGGTACATGGGCTCTGATTCCAAA
*chs1*	37	43213	chitin synthase	GAAAGAAGCGATAAAGTAGAGGCCGTAAATGGTAATC
*nsf1*	41	42584	general membrane fusion factor	CAACAGGGCATCGTCAATCATGTCTTTTCGATTCGTCATTC
*nth1*	43	44650	neutral trehalase	AACGTAACTGGCATTGACCCATCCAAATCCTTCTTTCGCAACG
*ctaA*	41	21112	ATPase copper transport	ACGAGTGATTGTGCCGGTTTTGTCCAAGACGACTTTGGTAA
**Pool3**				
*eno*	25	43415	enolase	TTACGGAAGTTGGTGCCAGCGTAGA
*rpl16a*	27	43406	ribosomal protein L13A, 60S subunit	CAACCTTCTTGCGCTCGTAGTAGGCAG
*gpd1*	29	20456	glyceraldehyde-3-phosphate dehydrogenase	ACGAAGTTGGGGTTCAGGGAGATACCAGC
*hem6*	29	13475	Coproporphyrinogen III oxidase, heme biosynthesis	ACTTCTTGAACCGAGGGTAGTACGTCTTG
*hsp70*	33	27833	heat shock protein	TTGGTGATGACAATCTTGTTGGACTTACCAGTG
*hsp105*	37	42578	ER chaperon	CGGGCTTATCCTCAGTGTCAACTTGTTGATAGAATAA
*rps16b*	35	29002	ribosomal protein S16A, 40S subunit	TGACACGGACGCGGATGTCGACGTTGGCGAACTTG
*acs1*	39	20403	acetyl CoA synthase	TTGTGCTTCTCAATAATGTCCCAGTACCTTGAGAAGTTG
*tps1*	41	21151	trehalose-6-phosphate synthase	AACTTGCGGATGAACTTGGTGATCCACGACTGGACATTCTG

Oligonucleotide probes binding to respective mRNA targets are organised into 3 pools according to their migration in capillary electrophoresis.
